# Higher plasticity in feeding preference of a generalist than a specialist: experiments with two closely related *Helicoverpa* species

**DOI:** 10.1038/s41598-017-18244-7

**Published:** 2017-12-19

**Authors:** Yan Wang, Ying Ma, Dong-Sheng Zhou, Su-Xia Gao, Xin-Cheng Zhao, Qing-Bo Tang, Chen-Zhu Wang, Joop J. A. van Loon

**Affiliations:** 1 The Institute of Chemical Ecology & College of Plant Protection, Henan Agricultural University, Zhengzhou, 450002 China; 2 Hengyang Normal University, Hengyang, 421008 China; 3 Institute of Plant Protection, Henan Academy of Agricultural Sciences, Zhengzhou, 450002 China; 4 State Key Laboratory of Integrated Management of Pest Insects and Rodents, Institute of Zoology, Chinese Academy of Sciences, Beijing, 100101 China; 5 Laboratory of Entomology, Wageningen University, Droevendaalsesteeg 1, 6708 PB Wageningen, The Netherlands

## Abstract

Herbivorous insects have been categorized as generalists or specialists depending on the taxonomic relatedness of the plants they use as food or oviposition substrates. The plasticity in host plant selection behavior of species belonging to the two categories received little attention. In the present work, fifth instar caterpillars of the generalist herbivore *Helicoverpa armigera* and its closely related species, the specialist *Helicoverpa assulta*, were fed on common host plants or artificial diet, after which their feeding preference was assessed individually by using dual - and triple- plant choice assays. Results show both the two *Helicoverpa* species have a preference hierarchy for host plants. Compared to the fixed preference hierarchy of the specialist *H*. *assulta*, the generalist *H*. *armigera* exhibited extensive plasticity in feeding preference depending on the host plant experienced during larval development. Whereas the specialist *H*. *assulta* exhibited a rigid preference in both dual and triple-plant choice assays, our findings demonstrate that the generalist *H*. *armigera* expressed stronger preferences in the dual-plant choice assay than in the triple-plant choice assay. Our results provide additional evidence supporting the neural constraints hypothesis which predicts that generalist herbivores make less accurate decisions than specialists when selecting plants.

## Introduction

Phenotypic plasticity can be defined as the ability of organisms to express modifications in morphology, physiology and/or behavior in response to fluctuating environments^1,2^. In an ecological context the degree of phenotypic plasticity of herbivores can be defined as their capacity to adapt to particular host plants, which is crucial to the understanding of evolutionary mechanisms, especially host plant shifts and sympatric speciation^3–6^.

Herbivorous insects have evolved different strategies to exploit their food sources that have been coined as generalism and specialism^7,8^. Even very closely related species of insects can vary significantly in diet breadth^9–11^. How different diet breadths evolved among closely related species is not well understood^3,12^. Integration of ecological and neurobiological approaches gave rise to the hypothesis that the evolution of diet breadth in animals is affected by ‘limited information’ or ‘neural constraints’, caused by limitations in the amount and rate of information processing in the brain^13–15^. This hypothesis has been studied mostly in vertebrates suggesting that attention constrains search behavior in a similar way across a broad range of species^16–22^. As for herbivorous insects, it predicts that generalist herbivores make slower and/or less accurate decisions than specialists when selecting hosts, because generalists must discriminate and decide among stimuli from a wider variety of potential hosts^14,23^. Comparative studies of generalists and specialists belonging to the same species or to sibling species allow testing of the ‘neural constraints’ hypothesis. Sibling species provide model systems for studies of evolution of diet breadth and its neural basis^9,24,25^. Thus far limited evidence has been reported for butterflies by Janz and Nylin^11^ and for aphids by Bernays and Funk^23^ that populations showing more specialized host selection behavior forage more efficiently than generalist populations. A direct comparison of host selection plasticity of caterpillars between specialist and generalist sibling species is lacking.

Herbivorous insects display a considerable degree of plasticity in host plant selection behaviors and this may have a significant effect on evolutionary change in host use^6,26–29^. For example, it has been well documented for several herbivorous insects that dietary experience with a particular compound^30–34^ or plant^27–29,35–37^ can increase the relative acceptability of that compound or plant. Such plasticity in host acceptance is an important factor in host specialization of herbivorous insects, and knowledge of host-choice plasticity is necessary in order to understand how host shifts occur and how diet breadth evolved^9,24,38,39^.

The cotton bollworm *Helicoverpa armigera* (Hübner) (Lepidoptera: Noctuidae) and the oriental tobacco budworm *Helicoverpa assulta* (Guenée) are closely related species and can be hybridized and backcrossed in the laboratory to produce viable offspring^40^. *H*. *armigera* is a true generalist and an agricultural pest that feeds on at least 161 plant species in 49 plant families, including cotton, sweet corn, tobacco, sunflower, hot pepper and so on^41,42^. In contrast, *H*. *assulta* is a specialist with a narrow host plant range, and mainly feeds on plant species in the Solanaceae such as tobacco, hot pepper, and several *Physalis* species^43^. Fifth instar caterpillars of *H*. *armigera* significantly preferred cotton leaf disks over pepper leaf disks, whereas caterpillars of *H*. *assulta* preferred pepper disks over cotton disks, and these contrasting preferences were consistent with the neural coding of the respective leaf saps by maxillary taste sensilla^10,25^.

In the present paper, we addressed the following inter-related questions: (1) Do caterpillars of both the generalist *H*. *armigera* and the specialist *H*. *assulta* display different feeding preferences? (2) Does exposure of early larval instars to different plant species induce plasticity of feeding preference in the two *Helicoverpa* species? (3) Are caterpillar feeding preferences of the two species consistent when comparing dual- and triple plant choice assays?

## Results

### Dual-choice leaf-disk assays on *H. armigera*

**Figure 1:**
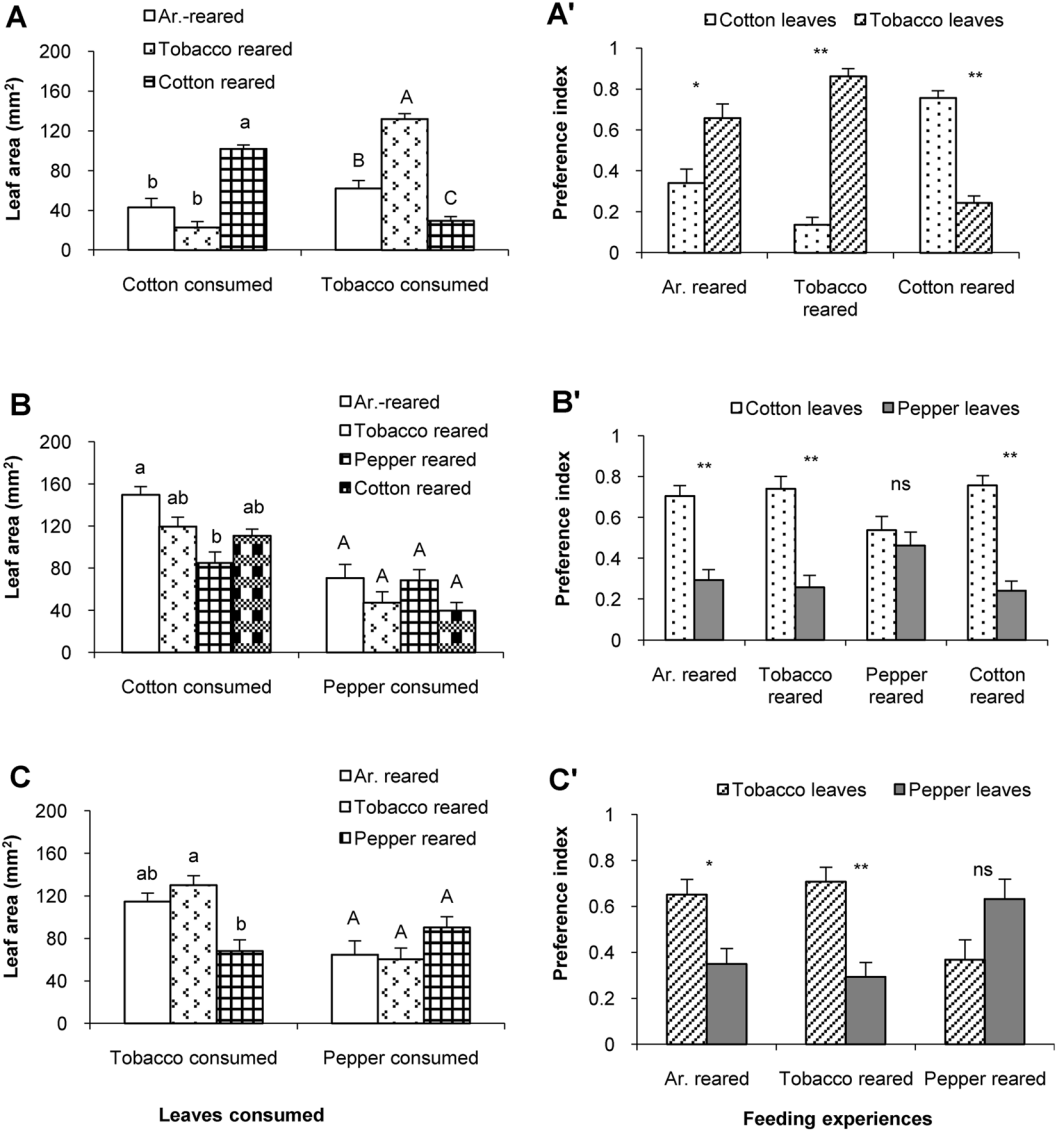
Feeding preference of *H*. *armigera* with different feeding experiences in dual-choice assay. Columns in (**A**), (**B**) and (**C**) represent the mean areas + SE of plant leaf disks consumed by caterpillars with different feeding experiences. Mean areas were compared by employing one-way ANOVA followed by Scheffé’s Post-Hoc test (*P* < 0.05). Columns having no lower case or capital letters in common differ significantly (*P* < 0.05). Columns in (**A**)’, (**B**)’ and (**C**)’ represent mean + SE of the preference indices for the 5^th^ instar caterpillars with the indicated feeding experience. The preference indices were compared in caterpillars with one feeding experience by employing the paired-sample t-test (*P* < 0.05). ‘*’ and ‘**’ represent the difference was significant at 0.05 and 0.01 level, respectively. ‘ns’: no significant preference different (*P* > 0.05).

Fifth instar caterpillars of the generalist *H*. *armigera* with different feeding experiences consumed different amounts in leaf choice assays (Fig. 1A). Mean leaf surface area of cotton consumed by cotton-reared caterpillars was 101.71 ± 6.90 mm^2^, which is significantly higher than that of artificial diet- and tobacco-reared caterpillars (Fig. 1A). Mean area of tobacco leaf consumed was 131.93 ± 5.33 mm^2^ in tobacco-reared caterpillars, which is significantly higher than that of artificial diet- and cotton-reared caterpillars (Fig. 1A), Tobacco consumed).

Feeding preference for cotton and tobacco leaves was compared between caterpillars of *H*. *armigera* which had been exposed to different diets from neonate to the 5^th^ instar (Fig. 1A’). Firstly, caterpillars reared on artificial diet significantly preferred to feed on tobacco leaf disks (P_T_ = 0.6591 ± 0.0689) rather than on cotton leaf disks (P_C_ = 0.3409 ± 0.0689) (df = 29, t = −2.541, *P* = 0.017) (Fig. 1A’, Ar. reared). Similarly, after exposure to the host plant tobacco, caterpillars significantly preferred tobacco leaf disks (P_T_ = 0.8633 ± 0.0370) rather than cotton leaf disks (P_C_ = 0.1367 ± 0.0370) (df = 29, t = −8.402, *P* < 0.001) (Fig. 1A’, Tobacco reared). Caterpillars of *H*. *armigera* raised on cotton leaves preferred cotton leaf disks over tobacco leaf disks (P_C_ = 0.7572 ± 0.0350; P_T_ = 0.2428 ± 0.0350) (df = 35, t = 7.255, *P* < 0.001) (Fig. 1A’, Cotton reared).

Leaf surface areas consumed were also significantly different in *H*. *armigera* caterpillars with different feeding experiences in the cotton-and –pepper choice assays (Fig. 1B). The mean cotton leaf areas consumed were similar in caterpillars reared on artificial diet, tobacco and cotton (Post-Hoc Scheffé’s Test after ANOVA: *P* = 0.472), whereas the mean areas of the cotton leaf consumed by pepper-reared caterpillars was significantly lower than that by artificial diet-reared caterpillars (Post-Hoc Scheffé’s Test after ANOVA: *P* = 0.015) (Fig. 1B, Cotton consumed). Caterpillars reared on the four diets consumed similar areas of pepper leaf in the cotton-and –pepper choice assays (Post-Hoc Scheffé’s Test after ANOVA: *P* = 0.263) (Fig. 1B, Pepper consumed).

Subsequently the feeding preference for cotton leaves and pepper leaves of caterpillars exposed to different feeding experiences was studied (Fig. 1B’). *H*. *armigera* caterpillars exposed to either of the three food sources, artificial diet, tobacco or cotton leaves, significantly preferred cotton leaf disks over pepper leaf disks (Artificial diet-reared: df = 15, t = 3.789, *P* = 0.002; Tobacco-reared: df = 24, t = 3.889, *P* = 0.001; Cotton-reared: df = 31, t = 5.217, *P* < 0.001) (Fig. 1B’). Exposure to pepper fruits, however, did not induce a preference for cotton leaf disks (P_C_ = 0.5383 ± 0.0678) over pepper leaf disks (P_P_ = 0.4617 ± 0.0678) (df = 28, t = 0.683, *P* = 0.500) (Fig. 1B’, Pepper reared).

Tobacco-reared *H*. *armigera* caterpillars consumed significantly higher amount of tobacco than pepper-reared caterpillars (Post-Hoc Scheffé’s Test after ANOVA: *P* = 0.030) (Fig. 1C, Tobacco consumed), whereas the amount of pepper consumed was similar in caterpillars with different feeding experiences (Post-Hoc Scheffé’s Test after ANOVA: *P* = 0.682) (Fig. 1C, Pepper consumed). Caterpillars of *H*. *armigera* that had experienced either artificial diet or tobacco leaves significantly preferred tobacco over pepper (Artificial diet-reared: df = 30, t = 2.302, *P* = 0.028; Tobacco-reared: df = 20, t = 3.254, *P* = 0.004) (Fig. 1C’, Ar.-reared and Tobacco -eared). Pepper fruit-reared *H*. *armigera* caterpillars did not prefer pepper leaf disks (P_P_ = 0.6321 ± 0.0863) over tobacco leaf disks (P_P_ = 0.3679 ± 0.0863) (Fig. 1C’, Pepper reared).

### Dual-choice leaf-disk assays on *H. assulta*

**Figure 2:**
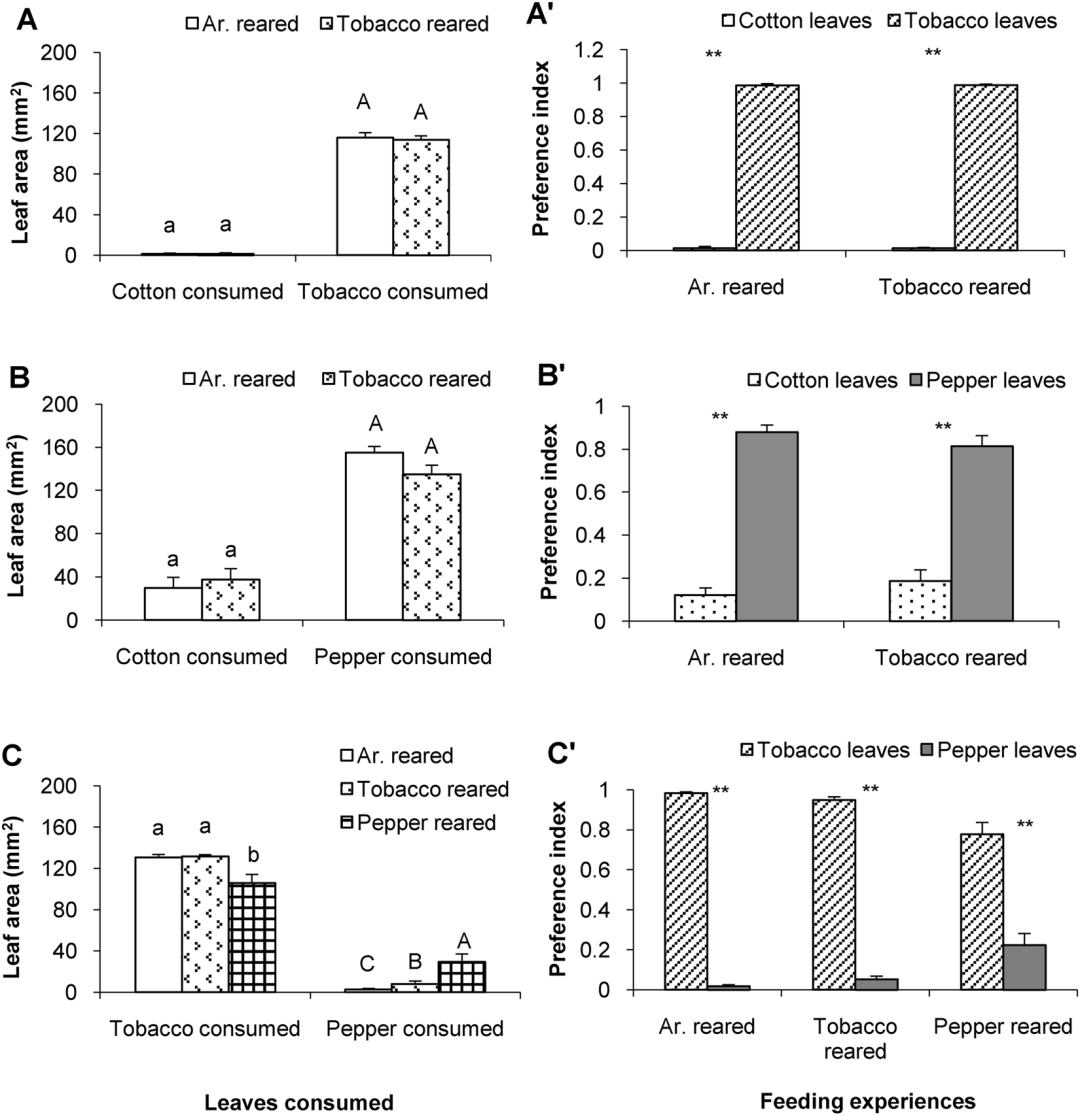
Feeding preference of *H*. *assulta* with different feeding experiences in dual-choice assay. Columns in (**A**), (**B**) and (**C**) represent the mean areas + SE of plant leaf disks consumed by caterpillars with different feeding experiences. Mean areas were compared by employing one-way ANOVA followed by Scheffé’s Post-Hoc test (*P* < 0.05). Columns having no lower case or capital letters in common differ significantly (*P* < 0.05). Columns in (**A**)’, (**B**)’ and (**C**)’ represent mean + SE of the preference indices for the 5^th^ instar caterpillars with the indicated feeding experience. Preference indices were compared in caterpillars with a certain feeding experience by employing the paired-sample t-test (*P* < 0.05). ‘*’ and ‘**’ represent the difference was significant at 0.05 and 0.01 level, respectively. ‘ns’: no significant preference different (*P* > 0.05).

Different from the generalist species *H*. *armigera*, the closely related specialist *H*. *assulta* exhibited less plasticity in its feeding preferences. Artificial diet- and tobacco-reared *H*. *assulta* caterpillars consumed similar amounts of cotton (t = −0.7435, df = 58, *P* = 0.4602) and tobacco (t = 0.1895, df = 58, *P* = 0.8503) in cotton *vs*. tobacco dual-choice assays (Fig. 2A). Caterpillars of *H*. *assulta* highly significantly preferred tobacco leaf disks after exposure to either artificial diet (P_T_ = 0.9865 ± 0.0100) or tobacco (P_T_ = 0.9880 ± 0.0061) (Ar-reared: df = 29, t = −37.014, *P* < 0.001; Tobacco-reared: df = 29, t = −44.441, *P* < 0.001) (Fig. 2A’).

Similarly, artificial diet-reared and tobacco-reared *H*. *assulta* caterpillars consumed similar amounts of cotton (t = 0.3325, df = 67, *P* = 0.7406) and pepper (t = 1.9446, df = 67, *P* = 0.0560) in the cotton *vs*. epper dual-choice assays (Fig. 2B). *H*. *assulta* caterpillars significantly preferred leaf disks of its host plant pepper over non-host cotton leaf disks after experience with either artificial diet or tobacco plants (Artificial diet-reared: df = 31, t = −10.859, *P* < 0.001; Tobacco-reared: df = 36, t = −5.990, *P* < 0.001) (Fig. 2B’).

Artificial diet-reared and the tobacco-reared *H*. *assulta* caterpillars consumed similar amounts of tobacco (Post-Hoc Scheffé’s Test after ANOVA: *P* = 0.998) (Fig. 2C). Pepper-reared caterpillars consumed the lowest amount of tobacco (Post-Hoc Scheffé’s Tests after ANOVA: *P* < 0.05) (Fig. 2C, Tobacco consumed) and the highest amount of pepper (Post-Hoc Scheffé’s Test after ANOVA: *P* < 0.01) (Fig. 2C, Pepper consumed).

In tobacco *vs*. pepper dual-choice assays caterpillars of *H*. *assulta* significantly preferred tobacco leaf disks over pepper leaf disks irrespective of feeding experience (artificial-diet-reared: P_T_ = 0.9824 ± 0.0176; df = 29, t = 38.641, *P* < 0.001; tobacco-reared: P_T_ = 0.9479 ± 0.0521; df = 29, t = 21.672, *P* < 0.001; pepper-reared: P_T_ = 0.7777 ± 0.0588; df = 28, t = 4.708, *P* < 0.001) (Fig. 2C’).

### Triple-choice assay on *H. armigera*

**Figure 3:**
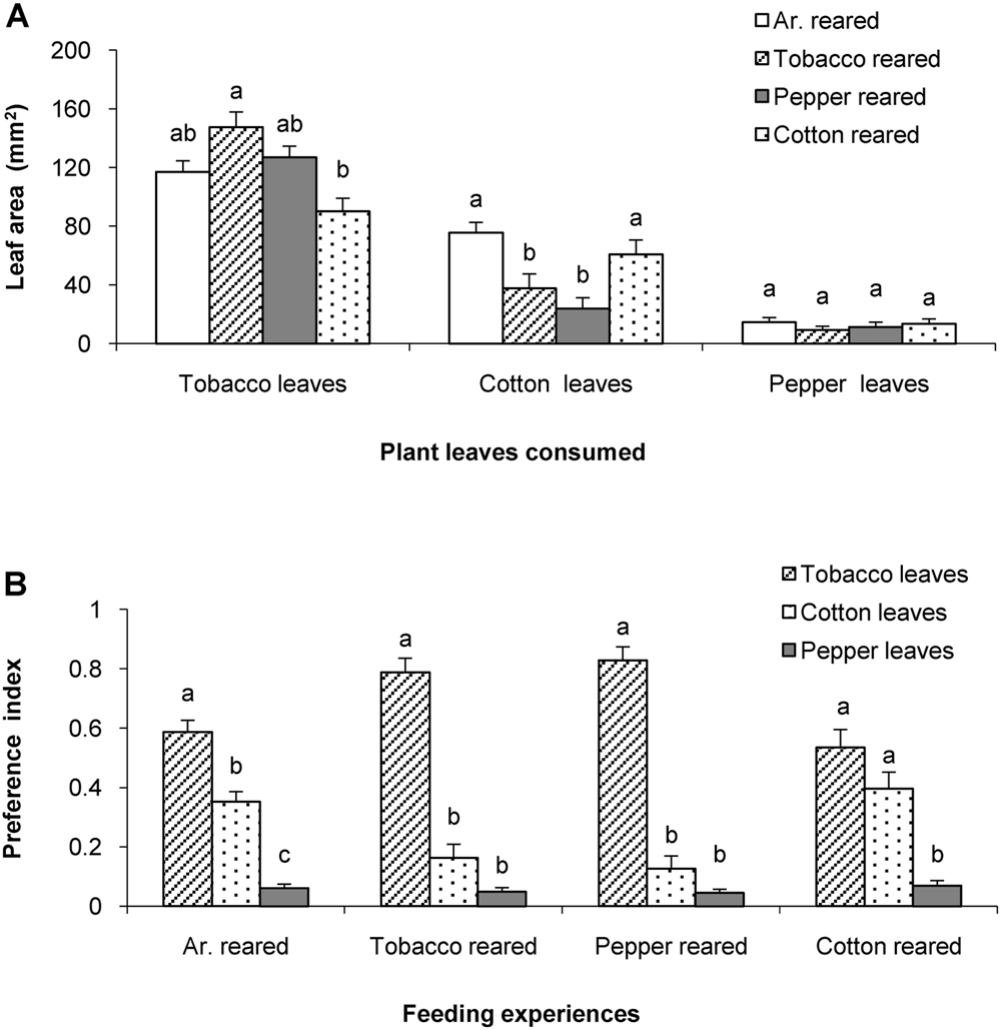
Feeding preference of *H*. *armigera* with different feeding experiences in triple-plant choice assays. (**A**) Consumed leaf areas by caterpillars belonging to one of four feeding experience groups. Columns in A represent the mean consumed leaf areas + SE of caterpillars with different feeding experiences. (**B**) Preference indices for three plant leaf species in caterpillars with one of four feeding experiences. Columns represent mean + SE of the preference indices for the 5^th^ instar caterpillars with the indicated feeding experience from neonates till the 5^th^ instar. Scheffé’s post hoc test for one-way ANOVA was used to compare the mean leaf areas and the preference indices (*P* < 0.05). Columns having no lower case letters in common differ significantly (*P* < 0.05).

The purpose of the triple-choice feeding assays was to assess whether caterpillars of the two *Helicoverpa* species had modified feeding preference when encountering more plant choices. Therefore, neonate caterpillars of *H*. *armigera* were reared on different diets until the early 5^th^ instar, and were then tested in triple-choice assays with tobacco leaf disks, cotton leaf disks and pepper leaf disks. Caterpillars of *H*. *armigera* with different feeding experiences ingested different amounts of leaf material. Tobacco-reared caterpillars consumed the highest and cotton-reared caterpillars the lowest amount of tobacco (Post-Hoc Scheffé’s Test after ANOVA: *P* = 0.031) (Fig. 3A, Tobacco leaves). Caterpillars reared on artificial diet and cotton ingested similar amounts of cotton (Post-Hoc Scheffé’s Test after ANOVA: *P* = 0.564) that were higher than that consumed by caterpillars reared on tobacco and pepper (Post-Hoc Scheffé’s Test after ANOVA: *P* = 0.993) (Fig. 3A, Cotton leaves). Caterpillars exposed to the four diets consumed similar an = mounts of pepper (Post-Hoc Scheffé’s Test after ANOVA: *P* = 0.883) (Fig. 3A, Pepper leaves).

The preference indices in choice assays with the three plants were compared in caterpillars reared on each of three diets. Caterpillars of *H*. *armigera* exposed to artificial diets mostly preferred to feed on tobacco leaf disks over cotton and pepper leaf disks (Fig. 3B, Ar.-reared: P_T_ = 0.5877 ± 0.0388; Post-Hoc Scheffé’s Test after ANOVA: *P* < 0.001), cotton was second in the preference hierarchy (Fig. 3B, Ar.-reared: P_C_ = 0.3514 ± 0.0342), while pepper was hardly fed upon (Fig. 3B, Ar.-reared: P_P_ = 0.0609 ± 0.0134; Post-Hoc Scheffé’s Test after ANOVA: *P* < 0.001).

After exposure to tobacco, the 5^th^ instar *H*. *armigera* also significantly preferred tobacco leaf disks over cotton and pepper (Fig. 3B, Tobacco reared: P_T_ = 0.7880 ± 0.04774; Post-Hoc Scheffé’s Test after ANOVA: *P* < 0.001), however, they did not discriminate among cotton and pepper (Fig. 3B, Tobacco reared: P_C_ = 0.1633 ± 0.0447; P_P_ = 0.0487 ± 0.0132; Post-Hoc Scheffé’s Test after ANOVA: *P* = 0.165). Similarly, caterpillars reared on pepper fruits also significantly preferred to feed on tobacco leaf disks over cotton and pepper leaf disks (Fig. 3B, Pepper reared: P_T_ = 0.7972 ± 0.0526; Post-Hoc Scheffé’s Test after ANOVA: *P* < 0.001), not discriminating between the two latter plants (Fig. 3B, Pepper reared: P_C_ = 0.1443 ± 0.0489; P_P_ = 0.0586 ± 0.0159; Post-Hoc Scheffé’s Test after ANOVA: *P* = 0.343). After caterpillars of *H*. *armigera* had been raised on cotton leaves, the 5^th^ instar larvae showed no preference for tobacco or cotton (Fig. 3B, Cotton reared: P_T_ = 0.5575 ± 0.05748; P_C_ = 0.3695 ± 0.05455; Post-Hoc Scheffé’s Test after ANOVA: *P* = 0.09), but preferred these over pepper (Fig. 3B, Cotton reared: P_P_ = 0.0730 ± 0.0170; Post-Hoc Scheffé’s Tests after ANOVA: *P* < 0.001 between P_P_ and P_T_, P_P_ and P_C_) (Fig. 3B, Cotton reared).

### Triple-choice assay on *H. assulta*

**Figure 4:**
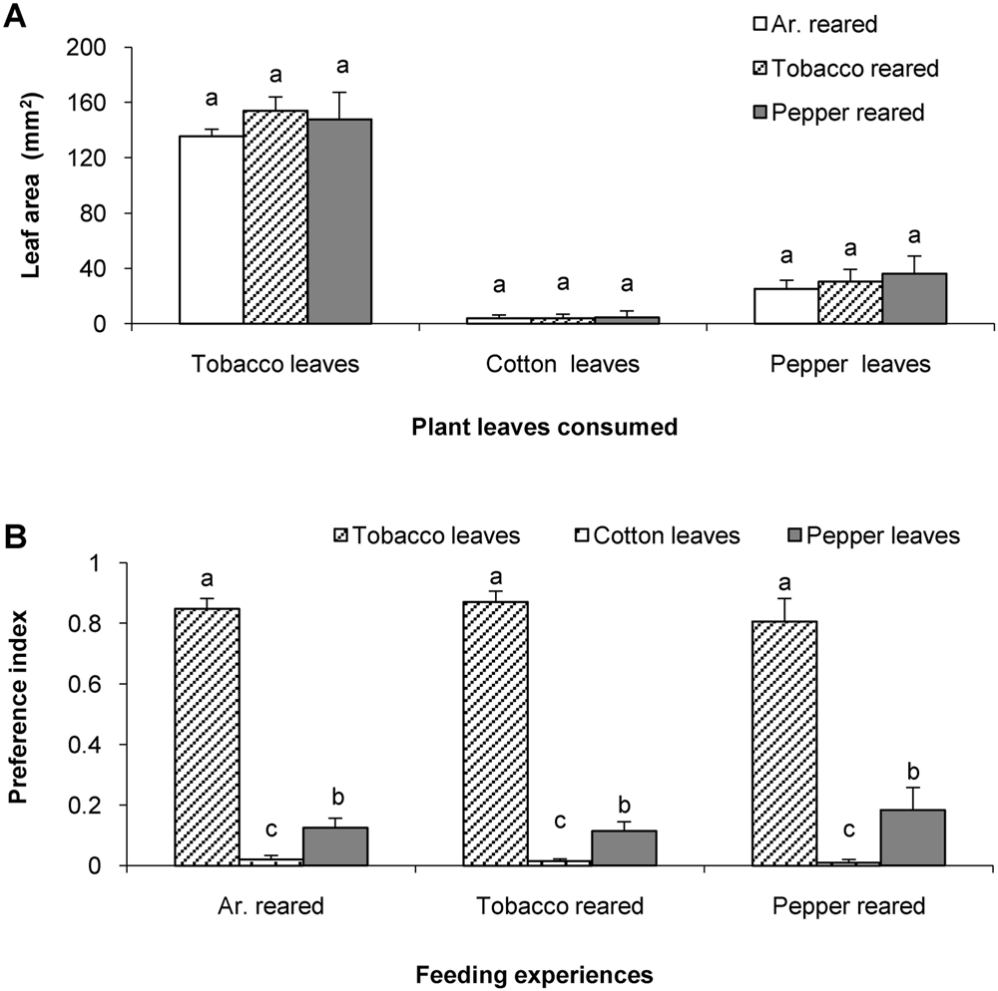
Feeding preference of *H*. *assulta* with different feeding experiences in triple-plant choice assay. (**A**) Consumed leaf areas by caterpillars that had one of three feeding experiences. Columns in (A) represent the mean consumed leaf areas + SE of caterpillars with different feeding experiences. (**B**) Preference indices for three plant species in caterpillars with one of three feeding experiences. Columns in (**B**) represent mean + SE of the preference indices for the 5^th^ instar caterpillars with the indicated feeding experience from neonates till the 5^th^ instar. Scheffé’s post hoc test for one-way ANOVA was used to compare the mean leaf areas and the preference indices (*P* < 0.05). Columns having no lower case letters in common differ significantly (*P* < 0.05).

Caterpillars of *H*. *assulta* exposed from neonate till the 5^th^ instar to tobacco, pepper fruits or artificial diet, respectively, were subjected to triple-choice assays in which tobacco leaf disks, pepper leaf disks and cotton leaf disks were offered. The consumed leaf areas for the same plant in *H*. *assulta* caterpillars with different feeding experiences were compared. The consumed leaf areas for tobacco leaves were similar in *H*. *assulta* caterpillars reared on artificial diet, tobacco and pepper (Post-Hoc Scheffé’s Tests after ANOVA: *P* = 0.833) (Fig. 4A, Tobacco leaves). Similarly, the consumed leaf areas of cotton were not significantly different in caterpillars with the three feeding experiences (Post-Hoc Scheffé’s Tests after ANOVA: *P* = 0.669) (Fig. 4A, Cotton leaves). The consumed leaf areas of pepper by caterpillars that had experienced pepper were also similar to those for the other two experience groups (Post-Hoc Scheffé’s Tests after ANOVA: *P* = 0.351) (Fig. 4A, Pepper leaves).

The results indicate that whatever the caterpillars had been exposed to, the 5^th^ instar larvae of *H*. *assulta* significantly preferred the host-plant tobacco (Ar.-reared: P_T_ = 0.8504 ± 0.0338; Tobacco-reared: P_T_ = 0.8716 ± 0.03544; Pepper-reared: P_T_ = 0.8066 ± 0.0761) (Post-Hoc Scheffé’s Tests after ANOVA: *P* < 0.001 in either plant or Ar.-reared caterpillars) (Fig. 4B). The host-plant pepper was second in the preference hierarchy (Post-Hoc Scheffé’s Tests after ANOVA: *P* < 0.001 between P_P_ and P_T_; *P* < 0.01 between P_P_ and P_C_ in either plant or diet-reared caterpillars), whereas leaf disks of the non-host cotton were the least preferred irrespective of feeding experience (Post-Hoc Scheffé’s Tests after ANOVA: *P* < 0.001 between P_C_ and P_T_; *P* < 0.01 between P_C_ and P_P_ in either plant or Ar.-reared caterpillars) (Fig. 4B).

### Comparisons of feeding preference in *H. armigera* caterpillars from wild and lab populations

**Figure 5:**
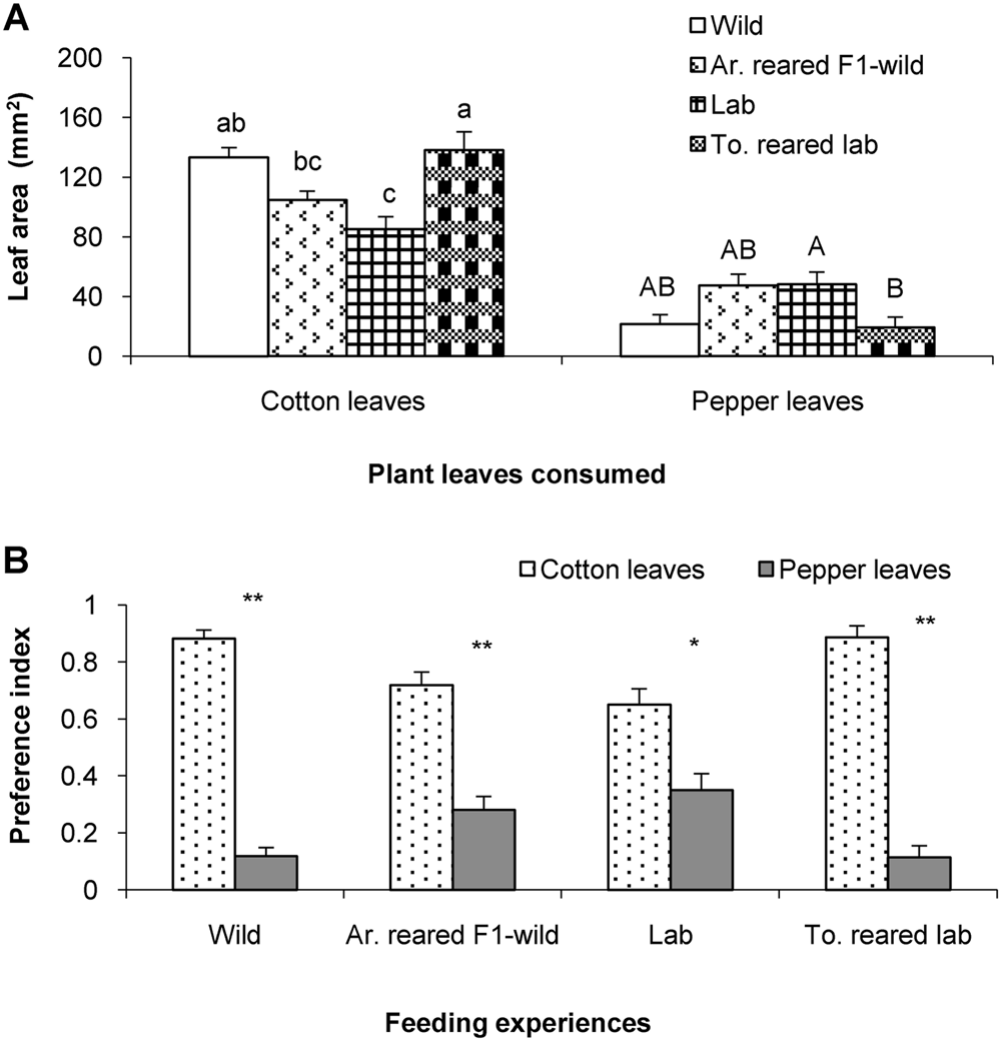
Feeding preference for cotton and pepper leaves in fifth instar caterpillars from different colonies. (**A**) Consumed leaf areas for the indicated plant species by caterpillars from different colonies. Mean areas were compared by using one-way ANOVA followed by Scheffé’s Post-Hoc test (*P* < 0.05). Different marks on columns indicate differences in preference for cotton leaves and pepper leaves (*P* < 0.05). Columns having no lower case or capital letters in common differ significantly (*P* < 0.05). (**B**) Preference indices for cotton and pepper leaf disks in caterpillars from the same colony by employing the paired-sample t-test (*P* < 0.05). Mark ‘ns’ represent the difference was not significantly different (*P* < 0.05), while mark ‘*’ and ‘**’ indicate the difference was significant at the 0.05 and 0.01 level, respectively. Wild: caterpillars directly captured from tobacco field; Ar.-Reared F1-wild: artificial diet-reared caterpillars of F1 generation derived from wild tobacco field; Lab: caterpillars reared by artificial diet in lab for at least ten generations; To. reared lab: tobacco-reared caterpillars derived from lab colony.

In order to investigate whether feeding preference of caterpillars differed between populations of different origin, we also compared the feeding preferences of *H*. *armigera* caterpillars obtained directly from the tobacco field (Wild), artificial diet-reared F1 generation derived from the field population (Ar.reared F1-wild), an artificial-diet based-lab colony (Lab) and a tobacco-reared lab colony (To.- reared lab). Firstly, the leaf areas consumed of the same plant were compared in caterpillars from different sources. Caterpillars from the wild population and from the tobacco-reared lab colony were consumed similar amounts of cotton (Scheffé’s Post-Hoc-Test: *P* = 0.983) and caterpillars from the Ar.-reared F1 generation and from the lab colony also consumed similar amounts of cotton (Scheffé’s Post-Hoc-Test: *P* = 0.417) (Fig. 5A, Cotton leaves). Caterpillars from the wild population consumed significantly higher amounts of cotton than those from the lab colony (Scheffé’s Post-Hoc-Test: *P* = 0.001) (Fig. 5A, Cotton leaves). Caterpillars from the wild population, its Ar.-diet-reared F1 and the lab colony consumed similar amounts of pepper (Scheffé’s Post-Hoc-Test: *P* = 0.121) (Fig. 5A, Pepper leaves). Caterpillars from all four groups significantly preferred cotton leaves over pepper leaves (*P* < 0.001 in Wild, Ar.-reared F1-wild and To.-reared lab; Fig. 5B; *P* = 0.015 in lab group) (Fig. 5B).

## Discussion

### Higher degree of plasticity in food preference in the generalist than the specialist

In nature, host plants of caterpillars are initially determined by the host plant selection of adult females. However, larvae are also known to develop well on some plant species which are not preferred by females as oviposition site^41,44–46^. Some species of larvae move between plant organs, *e*.*g*. leaves to reproductive structures and neighboring plants^45,47,48^. A new host plant species can be incorporated into an insect’s diet if the larvae accept it for feeding and are able to complete their life cycle on it^38,49,50^. The degree of plasticity in food preference of larvae is a relevant trait for host specialization^6,23,38^. Phenotypic plasticity in feeding preference behaviors in lepidopteran larvae has first been reported by Jermy *et al*.^51^ for the caterpillars of the specialist *Manduca sexta* and the generalist *Heliothis zea* that showed clear preferences for the plant to which they had been exposed. However, this study is the first to compare the plasticity of larval feeding preference between generalist and specialist sibling species.

The present study demonstrates that the generalist *H*. *armigera* was more plastic than its sibling specialist species *H*. *assulta* in its feeding preference after exposure to different diets. The generalist *H*. *armigera* exhibited obvious plasticity in host choices induced by various feeding experiences whereas the specialist *H*. *assulta* displayed a fixed preference hierarchy. Our findings demonstrate that the 5^th^ instar caterpillars of the generalist *H*. *armigera* developed a preference for the host plant on which the caterpillars were raised. For example, the tobacco-reared caterpillars significantly preferred to feed on tobacco leaves (Fig. 1A’, Tobacco reared), whereas the cotton-reared *H*. *armigera* caterpillars had a stronger preference for cotton leaves than tobacco leaves (Fig. 1A’, Cotton reared). It is known that caterpillars of *H*. *armigera* feed on cotton buds, cotton bolls and tobacco leaves while the 5^th^ instar is rarely observed to feed on cotton leaves in nature. A plausible explanation is that cotton leaves are less suitable food for *H*. *armigera* caterpillars compared to cotton buds and bolls. In our laboratory colony, the survival rate of cotton leaf-reared *H*. *armigera* caterpillars was substantially lower (51.70%) than that of tobacco-reared caterpillars (86.39%) and artificial diet-reared caterpillars (93.02%).

In contrast, the specialist *H*. *assulta* exhibited little plasticity in host-plant preference. Whatever the caterpillars of *H*. *assulta* were exposed to during the first four instars, the 5^th^ instar larvae of *H*. *assulta* decisively preferred tobacco either offered in a dual- or a triple-choice assay. Whereas pepper fruit-reared caterpillars of *H*. *armigera* did not discriminate between tobacco and pepper in the two-choice assay, *H*. *assulta* caterpillars raised on pepper still significantly preferred to feed on tobacco over pepper leaves in spite of the fact that pepper is a natural host plant of *H*. *assulta* (Fig. 2C’, Pepper reared, Fig. 4B, Pepper reared). Given a choice between the non-host plant cotton and the host plant pepper, *H*. *assulta* strongly preferred pepper leaf disks (Fig. 2B’). Therefore, irrespective of their feeding experience, *H*. *assulta* caterpillars most preferred tobacco, then pepper, and displayed a very low degree of acceptance of cotton leaves. Indeed, *H*. *assulta* did not survive if offered cotton leaves only. Consequently, the specialist *H*. *assulta* expresses a rigid host preference hierarchy that is not or only slightly modified by feeding experience and therefore seems genetically fixed. An indication for plasticity in preference was found in the tobacco-pepper choice assay; the preference indices for pepper obtained from caterpillars exposed to pepper fruits were significantly higher than those obtained from caterpillars exposed to artificial diet or tobacco (Fig. 2C, Pepper consumed) (Post-Hoc Scheffé’s Tests after ANOVA: *P* < 0.01).

### Differences in food consumption

Compared to the generalist *H*. *armigera*, caterpillars of *H*. *assulta* overall ingested substantially higher amounts of the preferred host plant in dual-choice assays (Fig. 2). In addition, food consumption of caterpillars also depended on their feeding experience, *e*.*g*. most artificial diet-reared *H*. *assulta* caterpillars did not feed on pepper leaves in the tobacco *vs*. pepper dual choice assays (Fig. 2C’, Ar.- reared). These results are consistent with the patterns in oviposition preference of the two sibling *Helicoverpa* species that showed that females of *H*. *assulta* were more sensitive than the *H*. *armigera* ^52^.

### Dual-choice vs. triple choice assays: consistent preference of the specialist, shifting preference of the generalist put into context of the neural constraints hypothesis

Feeding preference of the generalist *H*. *armigera* differed between dual-choice and triple-choice assays. Caterpillars of tobacco-reared *H*. *armigera* significantly preferred cotton leaves during the cotton-and-pepper dual-choice assay, whereas caterpillars with the same feeding experience did not discriminate the cotton and pepper leaves in the cotton-pepper-tobacco triple-plant choice assay. In addition, cotton-reared *H*. *armigera* larvae preferred cotton leaf disks in the tobacco-and-cotton two-choice assay, while caterpillars with the same feeding experience had similar preferences for tobacco and cotton leaves in the cotton-pepper-tobacco triple-plant choice assay. When plants belonging to four genera of the Asteraceae were offered, the generalist aphid *U*. *ambrosiae* settled on more suboptimal host plants than a specialist population, of which more than 60% settled on the preferred host plant *A*. *trifida* ^23^. These findings suggest that when more choices were offered, the specialists make more accurate choices than the generalists.

The present experiments also show caterpillars of *H*. *armigera* collected from a wild tobacco field and from the tobacco-reared lab colony were similar in their feeding preferences for cotton or pepper leaves, so were caterpillars collected from the artificial-diet-reared lab colony and from the artificial-diet-reared F1 generation derived from the field-collected population (Fig. 5). We conclude that the host plant preference of *H*. *armigera* caterpillars was affected by feeding experience but not by origin. For methodological reasons we used pepper leaves in our study instead of pepper fruits although the caterpillars had been reared on the fruits. The present study shows that caterpillars reared on pepper fruits had a higher preference for pepper leaves (Figs 1B’,C’; 2C), suggesting shared substances between pepper leaves and fruits that might induce the feeding preference for pepper leaves.

The neural constraints hypothesis states that specialist herbivorous insect species are better decision-makers than generalist species since the latter are neurally constrained in their ability to discriminate and decide among many potential hosts of different quality^14,53^. Better decisions mean that the preferred plants would bring fitness benefits. This hypothesis was supported by results of Janz and Nylin^11^ who found that specialist butterflies oviposited on plants supporting higher offspring survival, whereas generalist butterflies did not discriminate such higher-quality plants. Bernays^54^ demonstrated that the grasshopper *Schistocerca americana* reared as specialists initiated feeding faster after contact with food than those reared as generalists. Populations of the specialist aphid *Uroleucon ambrosiae* from eastern North America are highly specific to the host plant *Ambrosia trifida* (Asteraceae) and performed more efficiently in host-selection, host-acceptance and host-settling than the generalist populations from the American south-west with a variety of other taxa from the Asteraceae as hosts^23^. The present study supports the neural constraints hypothesis in the sense that caterpillars of the generalist *H*. *armigera* showed a less consistent feeding preference hierarchy than its specialist sibling *H*. *assulta* when comparing results of dual- and triple-choice assays.

### Neural mechanisms underlying host plant preference

The plasticity of feeding behavior induced by dietary exposure could be mediated by peripheral taste sensitivity, gustatory transduction pathways, centrifugal control by the central gustatory system or post-ingestive mechanisms^31,33,55,56^. The central projections of gustatory neurons in the sensilla styloconica displayed similar patterns in caterpillars of the two *Helicoverpa* species but differed in the distributions of neural branches in the subesophageal ganglion^57,58^. Molecular study using cDNA microarrays showed that the specialist *Manduca sexta* regulated transcripts in a diet-specific manner, while the generalist *Heliothis virescens* regulated a similar suite of transcripts across all diet types, suggesting that specialists are better adapted than generalist herbivores^59^. Our previous work on peripheral taste neurons of *H*. *armigera* indicated that neurons in the medial sensilla styloconica on the maxillary galea contribute to the gustatory discrimination between cotton leaf saps and pepper leaf saps, whereas taste neurons in the lateral sensillum of *H*. *assulta* contributed to the discrimination between cotton and pepper leaf saps^10,25^. Feeding experience affected gustatory sensitivity of the sucrose-best neuron in maxillary taste sensilla of *H*. *armigera* caterpillars exposed to three artificial diets differing in sucrose content^60^. Differences in sugar concentrations in the three food sources tested and the effects on sucrose-best neuron sensitivity may have contributed to the changes in food preferences displayed by *H*. *armigera* reported here.

Only recently has the possibility of different neuronal substrates underlying the taste plasticity of insects been considered. Genetic tools for monitoring and manipulating neuronal activity in the fruit fly *Drosophila melanogaster* promoted the understanding of the neural mechanisms of dopamine-mediated learning^61,62^. For example, dopaminergic neurons responsible for water learning are different from those for conveying the reinforcing effects of sugar. The dopamine-modulated neural circuits regulating naive water-seeking, learned water-seeking and water learning in *Drosophila* are also different in the brain of thirsty flies^63^. Dopaminergic neurons expressing the OAMB octopamine receptor specifically conveying short-term reinforcing effects of sweet taste^64^ were different from those for nutrient-dependent long-term memory by projecting to different regions in the mushroom body lobes^65^. In *Drosophila* two PPL1 subpopulations of dopamine neurons that innervate the α-lobe of the mushroom body were identified to be involved in aversive taste memory^62^. Future work could test if dopamine-modulated neural circuits are involved in the different degree of behavioral plasticity in response to dietary exposure we report here for the sibling *Helicoverpa* species.

## Conclusions

The present study demonstrates that the generalist *H*. *armigera* was more plastic than the closely related specialist species *H*. *assulta* in its feeding preference after exposure to different diets, suggesting that its host adaptation is more flexible than in the specialist. In addition, the generalist *H*. *armigera* exhibited a more variable feeding preference in the triple-plant choice assay than in the dual-plant choice assay, suggesting it performed more poorly if more choices were available. Our study is the first that compares the behavioral plasticity between sibling generalist and specialist herbivorous insects as induced by various feeding experiences. For future work, it will be relevant to examine the correlation between larval host-plant preference and oviposition preference. In addition, the gustatory mechanisms contributing to the behavioral plasticity could be investigated by comparing the gustatory sensitivity of caterpillars with different host-plant experiences.

## Materials and Methods

### Insects

Both caterpillars of *H*. *armigera* and *H*. *assulta* were obtained from established laboratory colonies, which were reared on an artificial diet^66,67^ prepared from the following ingredients: wheat bran (150 g), soybean powder (80 g), yeast powder (25 g), casein (40 g), sorbic acid (3 g), ascorbic acid (3 g), sucrose (10 g), agar (20 g), vitamin composite powders (8 g), acetic acid (4 ml), and distilled water (1500 ml). All colonies of the *Helicoverpa* caterpillars were maintained in the laboratory under controlled photoperiod (L16:D8) and temperature (27 ± 1 °C). Adults were supplied with a 10% v/v solution of sucrose in water.

**Figure 6:**
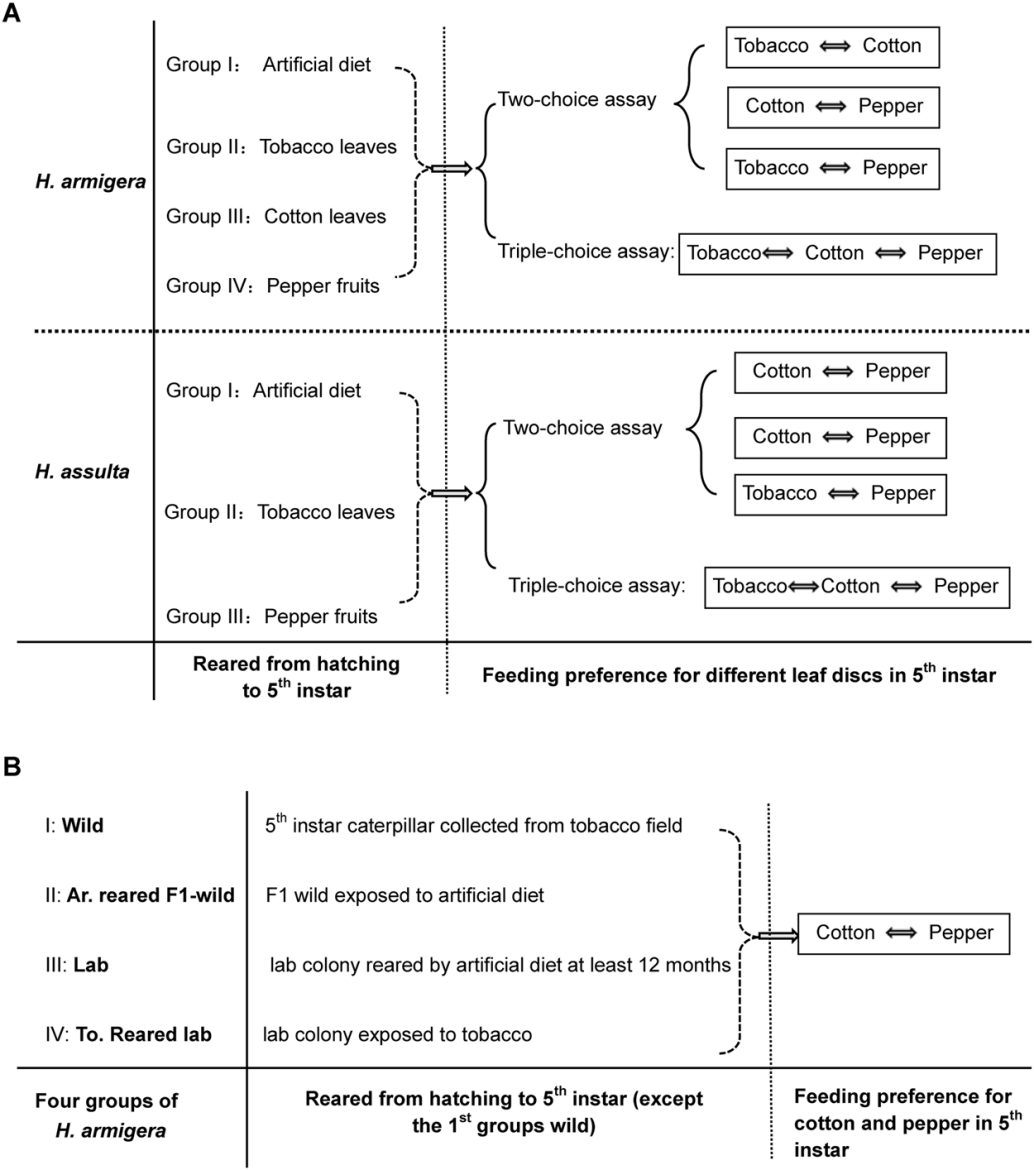
Overview of dietary experiences and the subsequent dual- and triple-choice leaf disk assays of the *Helicoverpa* caterpillars. (**A**) Caterpillars of the two *Helicoverpa* species originally collected from the lab colony reared on an artificial diet for at least 12 generations; (**B**) Caterpillars of *H*. *armigera* collected from tobacco field (Wild) and the lab colony (Lab), respectively.

Four groups of *H*. *armigera* caterpillars exposed to four different diets respectively were used in this experiment. Every group consisted of neonate caterpillars that were exposed to one diet until the fifth instar, the stage in which their feeding preference was tested individually (Fig. 6A). The four diets consisted of tobacco leaves, cotton leaves, hot pepper fruits and artificial diet. Among the four diets, tobacco and cotton are host plants of *H*. *armigera*, whereas hot pepper is not considered a favorite host plant of *H*. *armigera* since only rarely caterpillars of *H*. *armigera* have been found on hot pepper plants in nature and since feeding choice assays indicated that *H*. *armigera* avoids to feed on hot pepper^10^. In nature, caterpillars of *H*. *armigera* do feed on tobacco leaves, cotton buds and cotton bolls as well as tender cotton leaves before the third instar.

The specialist *H*. *assulta* was exposed to only three diets because neonates did not survive when forced to feed on cotton leaves. Therefore, tobacco leaves, hot pepper fruits and artificial diet were used to obtain respective groups of experienced *H*. *assulta* caterpillars (Fig. 6A). Tobacco and hot pepper are host plants of *H*. *assulta* and the artificial diet was the standard diet used to keep the lab colony of these species.

When feeding caterpillars with cotton or tobacco plants, fresh tender leaves of either species were put in a 12 cm-diameter glass Petri dish with a moist filter paper at the bottom. When pepper fruits were fed to caterpillars, fresh fruits were put in a food container (170 mm × 110 mm × 70 mm) with a moist paper at the bottom. After the third instar, only one caterpillar was put in a Petri dish or a food container to avoid cannibalism. Leaves or fruits were replaced by fresh ones each day.

We also tested and compared the feeding preference for cotton and pepper leaf disks of the 5^th^ instar caterpillars of *H*. *armigera* collected from the field and then exposed to artificial diet in the F1 generation as follows: (1) caterpillars directly from tobacco field (Wild); (2) neonates of wild population reared on artificial diet until the fifth instar (Ar. Reared F1-wild); (3) from established laboratory colonies fed on artificial diets (Lab); (4) neonates of established laboratory colonies reared on tobacco leaves until the fifth instar caterpillar (To. reared lab) (Fig. 6B).

### Plants

The plants used in the experiment were cotton *Gossypium hirsutum* L., “Zhong-12” (Malvaceae), a common host plant of *H*. *armigera* but non-host plant for *H*. *assulta*, pepper *Capsicum frutescens* L., “Nongyan-5” (Solanaceae), a host plant of *H*. *assulta* but not the favorite host plant for *H*. *armigera* in nature, and tobacco *Nicotiana tabacum* “K326” (Solanaceae), a host plant shared by the two *Helicoverpa* species. All plants were grown at the Modern Agricultural Sci-tech Demonstration Garden of Henan Agricultural University in a suburb of Zhengzhou, and watered as needed. Tobacco and cotton with five fully expanded true leaves were used for feeding exposure or leaf-disk choice assays. In this experiment, immature pepper fruits were used for feeding exposure whereas pepper leaves were used for feeding preference assays. The reasons that we did not use pepper leaves to rear *H*. *armigera* caterpillars were (1) most caterpillars could not survive till the 5^th^ instar if neonate caterpillars were exposed to pepper leaves. We used pepper leaves but not pepper fruits for the tests of feeding preference because for the other two tested plants, cotton and tobacco, leaves were also used for testing feeding preference (2) the flesh of pepper fruits was difficult to cut into slices similar to leaf disks; (3) the cut pepper fruits were quickly oxidising compared to leaves. Though food quality and quantity will likely have been different between pepper fruits and pepper leaves, the specific secondary metabolites belonging to Solanaceae which may play an important role in gustatory recognition are present both in pepper fruits and pepper leaves. Our previous work showed that artificial diet-reared *H*. *armigera* caterpillars preferred cotton leaves and *H*. *assulta* preferred pepper leaves in cotton *vs*. pepper dual choice assays, suggesting pepper leaves could be used to test the feeding preference of *Helicoverpa* caterpillars^10^. No chemical sprays were applied to the plants from two weeks before and during the experiments.

### Feeding choice bioassay

Dual-choice and triple-choice leaf disk bioassays were used to test the feeding preference of the two *Helicoverpa* caterpillars with different feeding experiences, aiming to compare host choice of the generalist and specialist species. The dual-choice leaf disk bioassay was conducted as described by Tang *et al*. (2006). In brief, four leaf disks (diameter 10 mm for each disk) punched from the leaves of either plant species, were arranged in an ABABABAB fashion around the circumference of the Petri dish (12 cm diameter) on moist paper. In the triple-choice leaf disk bioassay, 12 leaf disks, four of each of the three plant species were arranged in an ABCABCABCABC fashion around the circumference of the 12 cm-diameter Petri dish on moist paper.

Caterpillars to be tested were early fifth-instars and had been starved for about four hours. To start the test, a single caterpillar was placed in the middle of each dish and the leaf disk consumption was observed at approximately half-hourly intervals thereafter. All Petri dishes were put under evenly distributed fluorescent strip lights at a temperature of 27 ± 1 °C. Each assay was terminated when at least two of the four disks of either plant species (A or B or C) had been consumed (about 156 mm^2^), and then the disk areas of all plant disks consumed was measured using transparency film (PP2910, 3 M Corp.) with a 1 mm^2^ grid. Each caterpillar was tested only once. For the feeding preference assays at least 30 replicates were conducted. In general, it took three to five hours to finish the feeding of a single caterpillar. But few caterpillars finished the feeding in two hours and a few took six or more hours to finish the feeding.

### Data analysis

The leaf areas of each plant consumed were measured and the preference indices (P_C_, P_T_ and P_P_) were calculated for each caterpillar as follows:

Preference index for cotton (P_C_) = (cotton area consumed)/(area consumed of all plant disks)

Preference index for tobacco (P_T_) = (tobacco area consumed)/(area consumed of all plant disks)

Preference index for pepper (P_P_) = (pepper area consumed)/(area consumed of all plant disks)

The means of the leaf areas and the preference index for each plant species by caterpillars experienced with one of the plants were calculated. For comparing the means of the leaf areas of one plant species consumed by caterpillars with different feeding experiences, the data of the original leaf areas were transformed logarithmically and were compared by employing one-way ANOVA followed by Scheffé’s Post-Hoc test (*P* < 0.05). For comparing the feeding preferences for different plants in caterpillars with a certain feeding experience, the raw data of the preference indices were square-root-transformed and the paired-sample t-test was used to compare the means of the preference indices for two plant species in dual-choice assays (*P* < 0.05). By comparing the feeding preference of caterpillars for the three plant species in the triple-choice assay, one-way ANOVA was used to compare the preference indices, employing Scheffé’s Post-Hoc-Test for multiple comparisons (*P* < 0.05). All data were analyzed using SPSS software version 10.0.

## Electronic supplementary material

Dataset 1

Dataset 2

Dataset 3

Dataset 4

Dataset 5
